# Prevalence of HHV-6 Detection Among People Living with HIV: A Systematic Review and Meta-Analysis

**DOI:** 10.3390/v17040531

**Published:** 2025-04-05

**Authors:** Georgia Kostare, Evangelos Kostares, Michael Kostares, Perry N. Halkitis, Athanasios Tsakris, Theodoros Xanthos, Maria Kantzanou

**Affiliations:** 1 Department of Microbiology, Medical School, National and Kapodistrian University of Athens, 115 27 Athens, Greeceatsakris@med.uoa.gr (A.T.); maria.kantzanou@gmail.com (M.K.); 2 Department of Anatomy, Medical School, National and Kapodistrian University of Athens, 115 27 Athens, Greece; 3 Center for Health, Identity, Behavior, and Prevention Studies, School of Public Health (CHIBPS), Rutgers University, One Riverfront Plaza, Suite 1020, Newark, NJ 07102, USA; perry.halkitis@rutgers.edu; 4 Department of Biostatistics and Epidemiology, School of Public Health, Rutgers University, Piscataway, NJ 08854, USA; 5 School of Health Science, University of West Attica, 122 43 Athens, Greece

**Keywords:** human herpesvirus 6 (HHV-6), human immunodeficiency virus (HIV), AIDS, PCR, prevalence, meta-analysis

## Abstract

Human herpesvirus 6 (HHV-6) is a ubiquitous virus with significant implications for immunocompromised individuals, particularly people living with HIV (PLWH). This study aimed to estimate the prevalence of HHV-6 detection in blood samples among PLWH using molecular diagnostic techniques. A systematic literature search was conducted across multiple databases until September 2024, including studies that reported HHV-6 detection in blood samples of PLWH through molecular methods. The meta-analysis calculated pooled prevalence rates using a random-effects model and assessed study quality, with additional analyses for outlier identification and influential study effects. Twelve studies met the inclusion criteria, and the random-effects model estimated the prevalence of HHV-6 detection at 11.7% (95% CI: 4.3–21.8%), with considerable heterogeneity. Influence diagnostics identified one study as influential, and after its exclusion, the recalculated pooled prevalence was 8% (95% CI: 4.4–12.4%), with reduced but still considerable heterogeneity. This meta-analysis highlights the prevalence of HHV-6 detection in PLWH, emphasizing the need for ongoing research to explore the clinical implications and factors influencing viral detection as well as the implications of this coinfection on the treatment and overall health of PLWH.

## 1. Introduction

In total, 8 of the more than 100 herpesviruses that have been identified can infect humans. These include herpes simplex virus types 1 and 2 (HSV-1 and HSV-2), varicella-zoster virus (VZV), cytomegalovirus (CMV), Epstein–Barr virus (EBV), human herpesvirus 6 (HHV-6), human herpesvirus 7 (HHV-7), and Kaposi’s sarcoma-associated herpesvirus (KSHV or HHV-8) [1]. HHV-6, the sixth herpesvirus discovered, was first identified as the human B-lymphotropic virus. It was isolated from the blood lymphocytes of patients with lymphoproliferative diseases or acquired immunodeficiency syndrome (AIDS). It is now known that HHV-6A and HHV-6B are two distinct viruses that can cause latent, acute, and chronic infections. HHV-6A is more frequently detected in immunocompromised people, whereas HHV-6B causes roseola infantum (exanthem subitum), a self-limited pediatric disease characterized by a maculopapular rash on the neck and torso that disappears in a few days and a high fever that lasts two to five days. HHV-6B is responsible for nearly all primary infections in infants and is the main virus that reactivates in both healthy and immunocompromised individuals. Together, HHV-6A and HHV-6B infect more than 90% of individuals by age 2 to 5 years [2,3]. Following an initial infection, HHV-6 develops latency, primarily in mononuclear cells, and integrates into the host’s chromosomes in a unique way. Additionally, the virus survives in the salivary glands, and PCR can identify its DNA in saliva. The central nervous system (CNS) has been implicated as a site of latency or persistence by the discovery of HHV-6 DNA in the cerebrospinal fluid (CSF) of children during and after primary infection, as well as in the brain tissue of immunocompetent adults after death [4]. In addition, HHV-6 infection in infants has been associated with febrile status epilepticus and long-term neurological complications, reinforcing concerns about its impact on neurodevelopment in pediatric HIV cases. In immunocompromised individuals, including people living with HIV (PLWH), HHV-6 can act as an opportunistic pathogen. A recent CSF PCR study detected HHV-6 DNA in 1.9% of cases among individuals with CNS infections, often alongside other opportunistic pathogens such as CMV, VZV, and John Cunningham virus (JCV). This supports its potential role in HIV-related neurological disease. The frequent coexistence of multiple CNS infections highlights the value of molecular diagnostics for HHV-6 detection, though the routine implementation of CSF PCR remains limited in resource-constrained settings due to cost-effectiveness concerns [4,5].

The co-infection of HHV-6, particularly HHV-6A, with HIV-1 can have significant implications for HIV disease progression. Both viruses infect CD4+ T cells, and HHV-6A has been shown to accelerate HIV-1 expression and promote CD4+ T cell death, potentially exacerbating immune decline in PLWH. Experimental studies demonstrated that HHV-6 can productively co-infect human CD4+ T lymphocytes alongside HIV-1, leading to enhanced HIV-1 replication, increased viral antigen expression, and a synergistic cytopathic effect that accelerates CD4+ T-cell depletion. Additionally, HHV-6 transactivates the HIV-1 long terminal repeat (LTR), further amplifying HIV-1 expression and contributing to immune dysfunction. Reactivated HHV-6 has been shown to upregulate CCL5, which in turn recruits HIV target cells, providing more sites for viral replication. The virus also encodes virokines and viroceptors that modulate immune responses and facilitate persistent infection, further impairing host defenses [6,7].

Despite the experimental evidence of HHV-6’s ability to enhance HIV-1 replication and contribute to immune dysfunction, cohort studies have produced mixed results regarding its impact on HIV disease progression [6,8]. Some studies indicate no significant effect, while others suggest that co-infection with multiple herpesviruses, particularly HHV-8, may accelerate disease progression [6,9]. An analysis of HIV seroconverters revealed that, while univariate models identified associations between HSV-2, HHV-8, and HIV progression, only HHV-8 remained significant after adjusting for confounding variables [9]. Furthermore, individuals with four concurrent herpesvirus infections demonstrated an increased risk of developing AIDS, suggesting that cumulative viral burden may drive immune deterioration [6,8].

In contrast, pediatric populations, particularly infants with vertically acquired HIV, may be more susceptible to HHV-6-mediated disease exacerbation. Primary HHV-6 infection in HIV-positive infants has been linked to a more rapid disease course during the first year of life, highlighting its potential role in early immune decline. HHV-6 is also frequently detected in CSF and brain tissue, suggesting the CNS as a potential viral reservoir. In addition, primary HHV-6 infection in young children is a leading cause of febrile illness, febrile seizures, and emergency hospital visits, further underscoring its clinical relevance in pediatric HIV cases [10].

The introduction of highly active antiretroviral therapy (HAART) has significantly reduced the incidence of HHV-6 and CMV reactivation in AIDS patients, though the clinical implications of low-level HHV-6 persistence remain unclear. Even in the HAART era, CMV and HHV-6 viremia remain significant risk factors for immune activation and HIV disease progression. CMV viremia has been proposed as a better predictor of HIV disease progression than CD4+ T-cell count, emphasizing the need to monitor herpesvirus co-infections in PLWH. Given its ability to establish latency, reactivate in immunocompromised states, and modulate immune responses, HHV-6 remains an important virus of interest in the context of HIV infection [11].

Due to the lack of clinical or laboratory findings at initial assessment, diagnosing HHV-6 primary infection might be difficult. Except in cases of original infection, viremia is uncommon in healthy children. However, it can happen during reactivation in immunocompromised patients and occasionally in immunocompetent children infected with other β-herpesviruses. The ubiquity of infection, latency, and chromosomally integrated HHV-6 (ciHHV-6) confound diagnosis, making it challenging to distinguish between acute infection, latent infection, and ciHHV-6. Diagnostic techniques include direct nucleic acid amplification testing and serologic tests [1]. Passive maternal antibodies can mask seroconversion in infants, and variations in antibody levels from latent viral replication or cross-reactivity can make serologic diagnosis based alone on an increase in antibody titers deceptive [2,3]. As a result, the clinical diagnosis of primary infection is common. In complex cases, confirmation can be achieved through a combination of serologic evidence of new infection and the detection of HHV-6 DNA in serum or plasma via quantitative PCR. The detection of HHV-6 DNA alone is insufficient for diagnosing active infection, particularly in individuals with ciHHV-6 [1,3].

This article contributes to the field by presenting the results of a systematic review and meta-analysis that investigates the synergies between HHV-6 and HIV infection, specifically the prevalence of HHV-6 detection in PLWH. Despite the known role of HHV-6 in immunocompromised individuals, including transplant recipients, the detection of HHV-6 in PLWH remains underexplored. By addressing this knowledge gap, this study enhances our understanding of the prevalence of HHV-6 detection among PLWH and provides important insights that can guide future research and inform clinical monitoring strategies.

## 2. Materials and Methods

### 2.1. Search Strategy

Comprehensive searches were carried out in the Medline and PubMed Central (PMC) (via PubMed), Scopus, Web of Science, and partially in Google Scholar databases to capture the gray literature. This study followed the structure and reporting standards outlined in the Preferred Reporting Items for Systematic Reviews and Meta-Analyses (PRISMA) guidelines, and the PRISMA checklist is available in the Supplementary Materials (Supplementary Table S1). We included studies published up to 20 September 2024. Two independent reviewers conducted the literature search, utilizing a combination of keywords such as “HHV 6”, “HHV-6”, “HHV”, “Human herpesviruses”, “HIV”, “AIDS”, “immunodeficiency”, “immunosuppressed”, “HIV-positive”, “prevalence”, “incidence”, and “rate”. The full search algorithm for each database is available in the Supplementary Materials (Supplementary Table S2).

During the primary search, the reference lists of the identified studies were carefully reviewed to locate any additional articles that may have been missed. All identified studies were systematically organized and managed using Zotero reference management software (version 6.0.18). To ensure the reliability of the dataset, duplicates were meticulously removed.

Two separate researchers thoroughly examined the remaining publications following the initial search. There were two stages to the selection process: first, the abstracts and titles were evaluated, and publications that did not fit the predetermined inclusion criteria were disqualified. The remaining articles’ complete texts were acquired and carefully assessed in the second stage. Consistency and alignment in the decision-making process were ensured by the reviewers’ commitment to settle any differences during the selection process.

### 2.2. Criteria for Study Selection and Data Extraction

To guarantee clarity and precision in our systematic review and meta-analysis, we used the Population, Exposure, Comparison, Outcomes, and Study Types(PECOS) framework to construct our inclusion and exclusion criteria because this is a proportional meta-analysis.
**Eligibility Criteria****Inclusion Criteria****Exclusion Criteria**Population (P)PLWH who were evaluated for the detection of HHV-6Immunocompromised patients for reasons other than HIV infection and solely pediatric populationsExposure (E)The exposure of interest was the detection of HHV-6 in blood using molecular methodsOther human herpesviruses (HHVs)Comparison (C)Given that our objective was to quantify the prevalence, a direct comparison component does not apply within our study’s framework-Outcome (O)The primary outcome was the detection of HHV-6 in blood using molecular methods. Additionally, we aimed to identify risk factorsStudies assessing seropositivity using other diagnostic methods (e.g., serological methods such as ELISA), other body fluids (e.g., saliva, solely serum) [12,13,14], or different body tissuesStudy Type (S)Observational studies including cohort, case–control, and cross-sectional studiesCase reports, case series with ≤20 participants, review articles, systematic reviews, meta-analysis, interventional studies including randomized clinical trials and non-randomized clinical trials, comparative studies, animal studies, letters to the editor, books, expert opinions, conference abstracts, articles written in languages other than English, and studies without full-text availability

Data Extraction: For each included study, we collected the following information: the primary author’s name, publication year, study design, continent and country of origin, study duration, total number of PLWH, gender distribution, mean age, and the number of patients with detected HHV-6.

### 2.3. Quality Assessment

Two independent researchers thoroughly assessed each study using the Quality Assessment Tools created through a partnership between the Universities of Newcastle, Australia, and Ottawa, Canada. They used the modified Newcastle–Ottawa Scale (NOS) for cross-sectional studies and the original NOS for cohort studies. Finding any possible methodological or survey-related problems that would compromise the research’s internal validity was the goal of this study. Three main criteria were used to score the studies: the choice of study groups, the comparability of those groups, and the identification of exposure or outcome for case–control or cohort studies (or cross-sectional studies using the modified technique). This evaluation was conducted using a “star system”. Studies scoring between 7 and 9 were deemed low risk of bias (high quality), those with scores of 4 to 6 were rated as moderate quality, and scores from 0 to 3 indicated high risk of bias (low quality) [15].

### 2.4. Statistical Analysis

Statistical analysis and meta-analysis were conducted using R software (version 4.3.1) [16], with the metafor package [17] employed to estimate pooled prevalence and corresponding 95% confidence intervals (CIs) based on the DerSimonian and Laird random-effects model. The Freeman–Tukey double arcsine transformation was applied [18], and visual inspection of the forest plot, along with Cochran’s Q statistic and its *p* value, assessed heterogeneity among studies. The Higgins I^2^ statistic, indicating true heterogeneity magnitude, was calculated with its 95% CI, categorizing values into 0–40%, 30–60%, 50–90%, and 75–100% for not important, moderate, substantial, and considerable heterogeneity, respectively. Identifying influential outlying effect sizes involved screening for externally studentized residuals and leave-one-out diagnostics [19]. Meta-regression was not performed as variables such as mean CD4 cell count were excluded from the analysis due to insufficient data (fewer than ten studies) [20]. Unless specified otherwise, statistical significance was set at *p*  =  0.05 (two-tailed). To assess publication bias qualitatively in the context of comparative data, methods such as Egger’s test [21], Begg’s test [22], and funnel plots were often used. However, in this meta-analysis of proportions, there is a lack of clarity or consensus on defining positive results [23]. Consequently, these tests were not employed, and a qualitative approach was instead adopted for evaluating publication bias.

## 3. Results

### 3.1. Results and Characteristics of the Included Studies

**Figure 1:**
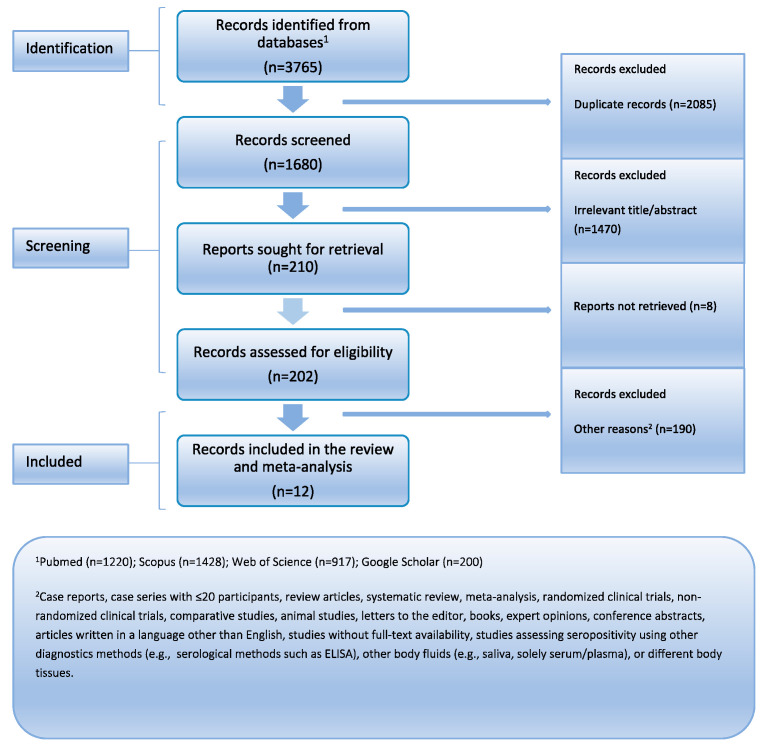
PRISMA flowchart.

Initially, 3765 records were identified across multiple databases. After removing duplicates, 1470 records were excluded based on irrelevant titles and abstracts. Full-text assessment led to further exclusions, resulting in the final selection of twelve eligible studies (totaling 1080 PLWH) for analysis. Figure 1 provides the PRISMA flowchart. Table 1 summarizes their descriptive characteristics. Every article was released in the years 1990–2020. Ten of the studies were cross-sectional, one was a case–control study, and one was a cohort study. Three investigations were carried out in North America (USA), one in Asia (Indonesia), one in Africa (Burkina Faso), six in Europe (UK, Germany, France, and Italy), and one in Australia. The mean age of participants ranged from 36 to 43 years (median: 37 years), and 75.8% of participants were male on average. All of the studies were assessed to be of moderate quality based on the quality assessment.

### 3.2. Prevalence of HHV-6 Detection Among PLWH

**Figure 2:**
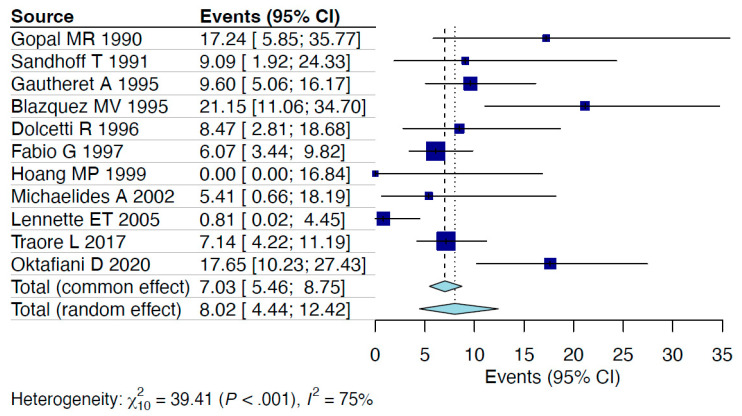
Forest plot [24,25,26,27,28,29,30,31,33,34,35].

A random-effects model analysis estimated the prevalence of HHV-6 detection among PLWH via molecular methods (including detection in peripheral blood mononuclear cells (PBMCs), peripheral blood leukocytes (PBLs), and whole blood) at 11.7% (95% CI: 4.3–21.8%), with considerable heterogeneity between studies (I^2^ = 90%, 95% CI: 88–98%, *p* < 0.001). Influence diagnostics and the forest plot illustrating the results of the leave-one-out analysis are presented in the Supplementary Materials (Supplementary Figures S1 and S2). According to these analyses, the study conducted by Fairfax MR et al. [32] was identified as influential. After excluding this study, the estimated prevalence was recalculated at 8% (95% CI: 4.4–12.4%), with less, still considerable remaining heterogeneity between studies (I^2^ = 75%, 95% CI: 50–93%, *p* < 0.001) (Figure 2).

## 4. Discussion

The present meta-analysis provides a comprehensive estimation of the prevalence of HHV-6 detection among PLWH through molecular methods. The overall pooled prevalence of HHV-6 detection in PBMCs, PBLs, and whole blood was estimated at 8% (95% CI: 4.4–12.4%), with considerable heterogeneity. This variability underscores the complexity of interpreting HHV-6 detection rates, particularly as it may reflect latent infections rather than active viral replication or the presence of chromosomally integrated HHV-6 (ciHHV-6). In PBMCs and PBLs, HHV-6 is often found in a latent state, where the virus persists without causing overt symptoms, although ciHHV-6 can also be detected in these cells as the viral genome is integrated into the telomeres of the host’s chromosomes. The reactivation of latent HHV-6, or the detection of ciHHV-6, can be triggered by immunosuppression, a common condition in PLWH, which might explain the detection of HHV-6 DNA in some studies, despite the absence of active infection markers. Whole blood samples, which may contain both a latent and actively replicating virus, further contribute to the complexity of interpreting these findings, particularly as they could also contain ciHHV-6 [36]. Regardless of these laboratory-based shortcomings, the clinical implications merit attention as to the potential synergies that may exist on the health of PLWH who are dually infected [37].

The significant heterogeneity observed across the analyses highlights the influence of various factors, including differences in molecular methods (e.g., sensitivity, targeted regions of the viral genome), geographic variations, characteristics of study populations, and types of samples used. Variability in detection rates is further impacted by the clinical status of PLWH, such as the level of immunosuppression. Although we aimed to conduct a meta-regression, fewer than 10 studies provided data on continuous variables, such as CD4 counts, age, or gender distribution, thereby limiting the scope of this analysis. Co-infections also likely contribute to the observed variability. Moreover, the absence of standardized protocols across studies amplifies this heterogeneity, as inconsistencies in sample processing, DNA extraction methods, and PCR protocols can markedly influence detection rates. The different periods in which these studies were conducted further compound this variability. It is also important to note that, in proportional meta-analyses, a high degree of heterogeneity is often expected. The high levels of heterogeneity also reflect the complex nature of HHV-6 infection and detection in PLWH. Latent HHV-6 may reactivate under varying clinical conditions, such as in cases of advanced immunosuppression or concurrent infections, leading to sporadic detection across different biological compartments. Moreover, the reactivation potential and clinical significance of HHV-6 in PLWH remain poorly understood, warranting further investigation into its role in immune dysfunction and disease progression. In patients infected with HIV, the impact of HHV-6 infection can be an up-regulator of HIV replication and accelerate progress towards AIDS [38,39]. Future research should explore the clinical implications of HHV-6 detection, focusing on its potential interactions with other viral infections and its impact on long-term health outcomes in PLWH.

Despite employing a random-effects model to account for between-study variability, the substantial heterogeneity observed in this meta-analysis suggests that the pooled prevalence estimates should be approached with caution. The small number of included studies reduces the robustness of the model, emphasizing the uncertainty surrounding the true prevalence of HHV-6 detection. Furthermore, the studies were conducted across various geographic regions, each with distinct epidemiological profiles and healthcare systems, potentially influencing detection rates and limiting the generalizability of the findings. The small sample sizes in many studies reduced statistical power, contributing to the observed variability. Moreover, the studies spanned several decades, during which significant advancements in diagnostic technologies and HIV treatments occurred, complicating direct comparisons between older and more recent studies. The lack of standardized diagnostic methods, particularly variations in PCR techniques and the use of different sample types, likely introduced methodological biases, exacerbating the heterogeneity. Additionally, fewer than ten studies reported variables such as CD4 counts, mean age, and percentage of males, limiting our ability to assess the role of potential confounding factors in HHV-6 detection through meta-regression. Another significant limitation was the exclusion of non-English language studies and those without full-text availability, potentially introducing selection bias. Moreover, our meta-analysis has not been registered in PROSPERO, which may be a source of bias. Additionally, some studies did not report viral load, which could have provided a more nuanced understanding of active versus latent infection. Furthermore, molecular diagnostics cannot distinguish between latent and acute infection. The high degree of heterogeneity suggests the need for more extensive, well-designed studies to yield more precise estimates and to clarify the clinical relevance of HHV-6 detection in PLWH. Finally, we also gathered studies that assessed HHV-6 detection among PLWH from serum/plasma samples. However, we identified only four studies, and the meta-analysis yielded extremely CI. Therefore, we decided not to include this analysis in the present study due to the uncertainty and reduced robustness of the model. Given these considerations, it is important to acknowledge that, like any meta-analysis, our study is subject to potential biases, including selection bias, language bias, search bias, heterogeneity bias, confounding bias, and data extraction bias, all of which may influence the validity and generalizability of our findings.

## 5. Conclusions

In conclusion, while this meta-analysis offers important insights into the prevalence of HHV-6 detection among PLWH, the considerable heterogeneity limits the generalizability of the findings. Future research should aim to address these limitations by employing larger study populations, and more homogeneous designs to provide more accurate estimates and clarify the clinical significance of HHV-6 detection among PLWH.

## Figures and Tables

**Table 1:** Detailed characteristics of the studies that were included in the evaluation.

First Author	Year of Publication	Study Design	Continent of Origin	Country	Study Period	PLWH	Proportion of Males (%)	Mean Age (years)	HHV-6 Positive	Positivity Rate (%)	Quality Assessment
Oktafiani D [24]	2020	Case–control	Asia	Indonesia	2016	85	NA	37	15	17.6	Moderate
Traore L [25]	2017	Cohort	Africa	Burkina Faso	2016–2017	238	33.2	NA	17	7.1	Moderate
Lennette ET [26]	2005	Cross-sectional	North America	USA	NA	123	NA	NA	1	0.8	Moderate
Michaelides A [27]	2002	Cross-sectional	Australia	Australia	1997–1998	37	100	43	2	5.4	Moderate
Hoang MP [28]	1999	Cross-sectional	North America	USA	NA	20	80	37	0	0	Moderate
Fabio G [29]	1997	Cross-sectional	Europe	UK	NA	247	NA	NA	15	6.1	Moderate
Dolcetti R [30]	1996	Cross-sectional	Europe	Italy	NA	59	NA	NA	5	8.5	Moderate
Gautheret A [31]	1995	Cross-sectional	Europe	France	NA	125	66	36	12	9.6	Moderate
Blazquez MV [32]	1995	Cross-sectional	Europe	Spain	1990–1993	52	NA	NA	11	21.1	Moderate
Fairfax MR [33]	1994	Cross-sectional	North America	USA	NA	32	100	36	24	75	Moderate
Sandhoff T [34]	1991	Cross-sectional	Europe	Germany	NA	33	NA	NA	3	9.1	Moderate
Gopal MR [35]	1990	Cross-sectional	Europe	UK	NA	29	NA	NA	5	17.2	Moderate

NA: Not applicable.

## Data Availability

Literature and Rstudio data are available from the corresponding author upon reasonable request.

## Supplementary Materials

The following supporting information can be downloaded at: www.mdpi.com/article/10.3390/v17040531/s1. Table S1: PRISMA checklist; Table S2: Literature search; Figure S1: Visual representation of the influence diagnostics for each of the included studies regarding the prevalence of HHV-6 detection among PLWH; Figure S2: Forest plot displaying the re-calculated pooled effects, with one study omitted each time, using the leave-one-out method.
